# Trigger finger presenting secondary to leiomyoma: a case report

**DOI:** 10.1186/1752-1947-3-7284

**Published:** 2009-05-08

**Authors:** Ziad Harb, Quamar Bismil, David M Ricketts

**Affiliations:** 1 St. George's Hospital, Blackshaw Road, Tooting, London SW17 0QT, UK; 2 South-West Thames Region, Commodore House, Juniper Drive, London SW18 1TZ, UK; 3 Princess Royal Hospital, Lewes Road, Haywards Heath, West Sussex RH16 4EX, UK

## Abstract

**Introduction:**

We present a previously undescribed entity: trigger finger secondary to a leiomyoma. This is the first time such a case has been reported and highlights the fact that common conditions can sometimes present secondary to rare diseases.

**Case presentation:**

A 39-year-old Caucasian man presented with a fairly typical presentation of trigger finger. During surgical treatment, the lesion was excised and sent for histology, which showed tissue consistent with a leiomyoma. The patient made an uneventful recovery.

**Conclusion:**

Trigger finger is a common condition that is usually easily diagnosed and managed. However, it is important to appreciate that uncommon conditions, such as leiomyoma, can present with what is sometimes considered trivial disease, and one should always consider the differential diagnoses even when faced with relatively benign conditions.

## Introduction

Trigger finger is a common condition, first described by Notta in 1850, characterised by painful clicking and locking of a digit. The underlying cause is a failure of normal tendon gliding in the A1 pulley region of the tendon sheath. This is usually associated with a proliferation of chondrocytes (stenosing tensosynovitis). We present a case of trigger finger secondary to a leiomyoma; a previously unreported condition.

## Case presentation

A 39-year-old, right-handed Caucasian male office worker presented with a three-month history of painful locking and clicking of his right ring finger. There was no history of trauma and he was otherwise fit and well. On examination, he had a tender mass on the volar aspect of the metacarpophalangeal joint as he actively flexed and extended the ring finger, and there was a small, palpable lump overlying the fourth metacarpophalangeal joint.

A diagnosis of trigger finger was made; however, the palpable lump was significantly larger than usually found with a Notta's nodule. An X-ray of the right hand was unremarkable and an ultrasound scan revealed an elliptical soft tissue mass associated with the flexor tendons and A1 pulley.

**Figure 1:**
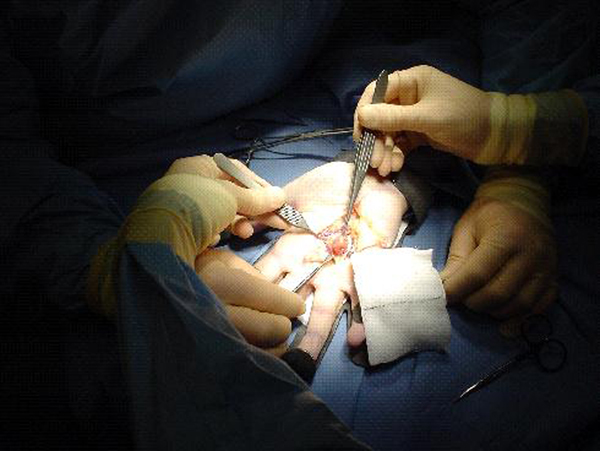
**Intraoperative photograph of the leiomyoma**.

At surgical exploration under general anaesthesia, a circumscribed soft tissue mass, 1.5 cm in diameter, was found infiltrating the A1 pulley and flexor digitorum superficialis (FDS) (Figure 1). The lesion was excised en masse, protecting the neurovascular bundle, and the postoperative course was uneventful. At six-week follow-up the wound had healed and the patient reported that the painful locking and clicking had completely resolved. The period of follow-up is now over 18 months and he has returned to his normal work and leisure activities without any further problems with the finger.

Histological examination of the excised tumour revealed a spindle-celled stromal lesion with mucinous degeneration; and with cells that were positive for SMAC (smooth muscle actin) on immunohistochemical analysis. These findings were consistent with a leiomyoma.

## Discussion

Trigger finger, also known as stenosing tenosynovitis, is seen in healthy middle-aged women and younger patients with occupations that involve repetitive and prolonged gripping and grasping. It is also associated with other conditions such as rheumatoid arthritis, gout, amyloidosis and diabetes; in these cases it is referred to as secondary trigger finger.

Rarely, trigger finger is caused by space occupying lesions or anatomical abnormalities which compromise tendon gliding. Previously described examples include: phalangeal exostosis [1]; anomalous lumbrical insertion [2]; giant cell tumour of the tendon sheath [3]; and lipoma [4].

Although the exact aetiology of trigger finger is commonly unknown, the majority of cases are related to a fusiform swelling of the flexor tendon at the level of the A1 pulley in the region of the metacarpophalangeal joint. The swelling in the tendon is often due to repetitive trauma which leads to inflammation, fibrosis and thickening of the tendon; or it can be due to the healing process after direct trauma or laceration. This nodule compromises the normal gliding of the tendon thorough its sheath, thus leading to the symptoms of snapping, triggering or locking. In our patient, a leiomyoma was found to be compromising tendon gliding in the region of the A1 pulley.

Benign tumours are common in the hand, but leiomyoma is uncommon: in a series by Butler et al of 437 hand tumours only one was a leiomyoma [5]. Leiomyoma is a benign proliferation of smooth muscle mesenchyme, is commonly well-differentiated, and hardly ever transforms to a malignant tumour. Leiomyomata can arise wherever there is smooth muscle tissue, and the anatomical site of the tumour determines the different subtypes of leiomyoma: for example in the skin (referred to as piloleiomyomas); in blood vessels (i.e. angioleiomyomas); and within the dartos muscle of the scrotum, the labia majora, or the erectile tissue of the nipple, collectively classified as genital leiomyoma. To our knowledge, there are no previous reports in the literature of a leiomyoma presenting with a triggering digit.

As we demonstrate with this case, uncommon pathology may present in a relatively common condition. We advise careful and methodical assessment of patients presenting with trigger finger, because although the majority of cases will be routine and uncomplicated, there are rare occasions when an underlying tumour is the cause of symptoms. If there are any doubts about the possible aetiology, then further investigations such as ultrasonography or magnetic resonance imaging should be utilised.

## Consent

Written informed consent was received from the patient for this publication. A copy of the written consent is available for review by the Editor-in-Chief of this journal.

## Competing interests

The authors declare that they have no competing interests.

## Authors' Contributions

ZH and QB wrote and revised the manuscript. DR was involved in the operative management and overall care of the patient, and provided guidance throughout the preparation of the manuscript.
